# Dose-dependent hepatorenal toxicity of polystyrene microplastics in BALB/c mice

**DOI:** 10.1371/journal.pone.0362569

**Published:** 2026-11-12

**Authors:** Samia Iftikhar, Hassien M. Alnashiri, Mehnaz Akhtar, Zuniar Ahsan, Muhammad Latif, A. El-Shabasy, Mohamed Mostafa Soliman

**Affiliations:** 1 Department of Zoology, Division of Science and Technology, University of Education, Lahore, Pakistan; 2 Department of Biology, College of Science, Jazan University, Jazan, Saudi Arabia; 3 College of Animal Science and Technology, Yangzhou University, Yangzhou, China; 4 Department of Zoology, Faculty of Science, Ain Shams University, Cairo, Egypt; Jiangsu University, CHINA

## Abstract

Microplastics have emerged as pervasive environmental contaminants with increasing concern regarding their potential adverse effects on mammalian health. However, comprehensive evaluations of the dose-related hepatorenal toxicity of polystyrene microplastics (PS-MPs) remain limited. This study investigated the effects of oral exposure to PS-MPs on hepatic and renal function in BALB/c mice. Animals were randomly assigned to four groups (n = 10/group) and orally administered 0 (control), 10, 20, or 30 mg PS-MPs/kg body weight/day for 28 consecutive days. Hepatorenal toxicity was evaluated using serum biochemical biomarkers, histopathological examination, semi-quantitative lesion scoring, and Pearson’s correlation analysis. PS-MP exposure produced dose-related increases in serum alanine aminotransferase (ALT; *P* = 0.0247), aspartate aminotransferase (AST; *P* = 0.0192), alkaline phosphatase (ALP; *P* = 0.0044), bilirubin (*P* = 0.0045), creatinine (*P* = 0.0029), and urea (*P* = 0.0006), with generally higher values observed at increasing PS-MPs doses indicating hepatic and renal dysfunction. Histopathological assessment demonstrated increasing severity of hepatic inflammatory infiltration, hepatocellular degeneration, necrosis, sinusoidal dilatation, and renal tubular epithelial degeneration/necrosis with increasing PS-MP dose. Semi-quantitative histopathological scores differed significantly among groups (Kruskal–Wallis, *P* < 0.0001), with the greatest lesion severity observed in the high-dose group. Pearson’s correlation analysis revealed significant positive correlations between creatinine and urea (*r* = 0.7628, *P* < 0.0001) and between ALP and bilirubin (*r* = 0.5229, *P* = 0.0087), supporting the biochemical evidence of renal and hepatobiliary dysfunction. In conclusion, oral exposure to PS-MPs was associated with hepatorenal toxicity,characterized by biochemical alterations and increasing histopathological injury with increasing exposure dose. These findings provide experimental evidence of the adverse effects of PS-MPs on mammalian liver and kidney function and contribute to a better understanding of the potential health risks associated with chronic microplastic exposure.

## 1. Introduction

Plastic pollution has become one of the most pressing environmental challenges due to the extensive production, persistence, and accumulation of synthetic polymers in terrestrial and aquatic ecosystems [1,2]. Among these contaminants, microplastics (MPs), defined as plastic particles smaller than 5 mm, have attracted increasing attention because of their widespread distribution and potential adverse biological effects [3,4]. Generated through the fragmentation of larger plastic debris, MPs are now detected in food, drinking water, soil, and air, creating multiple pathways for human and animal exposure [5,6].

Polystyrene (PS) is among the most abundant and frequently detected microplastic polymers because of its widespread use in packaging, disposable consumer products, and industrial applications [4]. Owing to their small particle size, large surface area, and high chemical stability, PS-MPs can readily interact with biological systems following ingestion and may cross physiological barriers [5,7]. Experimental evidence indicates that PS-MPs accumulate in mammalian tissues, including the gastrointestinal tract, liver, and kidneys, where they induce cellular dysfunction and tissue injury [8].

The liver and kidneys are primary targets of xenobiotic toxicity because of their essential roles in metabolism, detoxification, filtration, and excretion [9]. Following exposure, PS-MPs can enter the systemic circulation and accumulate in these organs, inducing oxidative damage and cellular dysfunction that impair tissue integrity [8,10]. Experimental studies have reported that PS-MP exposure can alter hepatic metabolic processes and antioxidant defenses, while renal effects have been associated with changes in tubular function and cellular signaling [10,11]. In particular, inflammatory and mitochondrial responses have been implicated in PS-MP-associated renal injury [12]. These findings indicate that the liver and kidneys may be important organs for evaluating the biological effects of PS-MP exposure.

Oxidative stress is an important mechanism involved in toxicant-induced tissue damage, as chemicals such as DMBA and CCl_4_ can disrupt antioxidant defenses and promote cellular and histopathological alterations [13–15]. Synthetic organoselenium compounds have demonstrated protective antioxidant effects against chemically induced damage in rat liver, lung, kidney, erythrocytes, mammary tissue, heart, and adrenal tissue [16,17]. Similarly, propolis and grape seed extract have shown protective effects against experimentally induced tissue injury through antioxidant and related cellular pathways [15,18,19]. Such evidence highlights the importance of oxidative imbalance and antioxidant defense in toxicant-associated tissue alterations and provides a broader context for understanding the potential biological effects of environmental contaminants, including PS-MPs.

Although considerable progress has been made in understanding microplastic toxicity, important knowledge gaps remain regarding the effects of PS-MPs on mammalian organs [20]. Existing studies have largely examined acute or single-dose exposures and often focused on isolated biochemical or histopathological endpoints [21]. Consequently, integrated evaluations of PS-MP exposure combining serum biomarkers with histopathological assessment of both hepatic and renal injury are still scarce [8,12]. Evaluating serum biochemical markers together with tissue histopathology may therefore provide a more comprehensive assessment of PS-MP-associated hepatorenal changes.

Accordingly, this study investigated the dose-related toxic effects of orally administered PS-MPs in BALB/c mice. BALB/c mice received graded oral doses of PS-MPs (10, 20, or 30 mg PS-MPs/kg body weight/day) for 28 days. Hepatorenal toxicity was subsequently evaluated using serum biochemical markers (ALT, AST, ALP, bilirubin, urea, and creatinine) and histopathological analysis. We hypothesized that repeated PS-MP exposure would induce dose-related hepatorenal toxicity, manifested by biochemical dysfunction and histopathological alterations.

## 2. Materials and methods

### 2.1. Study design and ethical approval

This experimental animal study was conducted in the Department of Zoology, University of Education, Lahore, Pakistan, to investigate the hepatotoxic and nephrotoxic effects of orally administered PS-MPs in mice. The experimental protocol was approved by the Campus Ethical Committee, University of Education, Lahore (Approval No. UE/MUL/CEC/008). All animal handling and experimental procedures were performed in accordance with institutional guidelines for the care and use of laboratory animals. The study was conducted and reported in accordance with the ARRIVE 2.0 guidelines for animal research wherever applicable [22].

### 2.2. Experimental animals and housing

Forty healthy adult male BALB/c mice (*Mus musculus*)**,** aged six weeks, weighing 25–30 g (average body weight approximately 27 g), were obtained from the Veterinary Research Institute (VRI), Lahore, Pakistan. Animals were acclimatized for one week before the experiment under controlled laboratory conditions (22 ± 2 °C, 50–60% relative humidity, and a 12 h light/12 h dark cycle) with free access to standard laboratory pellet diet and clean drinking water. Animals were observed daily for clinical signs of toxicity, including alterations in behavior, food and water intake, body condition, and mortality throughout the experimental period.

Throughout the 28-day experimental period, animals were monitored at least once daily for general health and clinical signs of pain or distress. Clinical observations included body condition, posture, grooming behavior, locomotor activity, food and water intake, responsiveness to external stimuli, and mortality. Body weight and clinical observations were additionally recorded on days 0, 7, 14, and 28. Humane endpoints were established before the initiation of the study. Animals exhibiting severe or persistent signs of distress, including body weight loss exceeding 20% of baseline, inability to eat or drink, persistent recumbency, severe dehydration, marked lethargy, or any condition indicating unnecessary suffering, were to be humanely euthanized immediately. No animals reached the predefined humane endpoints, no animals required early euthanasia, and no animals died unexpectedly during the study. All animals completed the planned 28-day experimental protocol.

### 2.3. Chemicals and reagents

The physicochemical characteristics of the PS-MPs were based on the manufacturer’s specifications. The particles consisted of uniform spherical polystyrene microspheres with a nominal diameter of 5.0 μm (Product No. 6-1-0500; Delivery Batch No. YJ202512223562; Tianjin BaseLine ChromTech Research Centre, China). The particles were supplied as dry powder and stored at 4°C prior to use. Fresh PS-MP suspensions were prepared immediately before each administration and sonicated in an ultrasonic bath (Model GS0203; 40 kHz frequency, 120 W ultrasonic power) for 25 min in continuous mode. The bath temperature was maintained at approximately 26 °C. To prevent particle settling and ensure uniform dispersion, the suspensions were vortexed thoroughly immediately prior to each administration and drawn into the syringe/gavage needle while maintaining suspension agitation during dosing.

### 2.4. Experimental grouping

Following acclimatization, mice were randomly allocated into four experimental groups (n = 10 per group) using a computer-generated randomization sequence. The control group received distilled water only, whereas the experimental groups received PS-MPs suspended in distilled water at doses of 10 mg PS-MPs/kg body weight/day (low dose), 20 mg PS-MPs/kg body weight/day (medium dose), and 30 mg PS-MPs/kg body weight/day (high dose) by oral gavage once daily for 28 consecutive days**.** Fresh PS-MP suspensions were prepared immediately before each administration by dispersing the particles in distilled water to obtain a homogeneous suspension. The gavage volume was maintained at 10 mL/kg body weight**,** which is within the recommended volume for oral administration of aqueous suspensions in mice, and was adjusted according to the body weight of each animal. Body weight and clinical observations were recorded on days 0, 7, 14 and 28.

### 2.5. Blood and tissue collection

At the end of the experimental period, mice were weighed individually and all animals across all experimental and control groups were anesthetized using the same standardized protocol by intraperitoneal injection of ketamine (100 mg/kg) and xylazine (10 mg/kg) (Flecknell, 2015)**.** Adequate anesthesia was confirmed by the absence of the pedal withdrawal reflex before sample collection. Blood samples were collected by terminal cardiac puncture and allowed to clot at room temperature. Following blood collection, samples were centrifuged at 3000 rpm for 10 min to obtain serum. The separated serum was transferred into labeled microcentrifuge tubes and analyzed fresh on the same day without undergoing any freeze-thaw cycles. Following terminal cardiac puncture, animals were euthanized by cervical dislocation under deep anesthesia in accordance with the approved institutional animal care protocol. Ketamine/xylazine can transiently influence hemodynamics and biochemical parameters; therefore, all groups (control and treatment) were sampled under identical timing and anesthetic conditions to minimize inter-group variability. Liver and kidney tissues were immediately excised, rinsed gently with ice-cold phosphate-buffered saline (PBS), blotted dry, and fixed in 10% neutral buffered formalin for 24–48 hours before histopathological examination.

### 2.6. Biochemical analysis

Serum biochemical parameters were measured using a Microlab 300 semi-automated clinical chemistry analyzer (VitalScientific, Netherlands) according to the manufacturers protocols. Prior to sample testing, instrument calibration was performed using dedicated standard calibrators, and internal quality control samples (low and high control levels) were analyzed to ensure assay accuracy, precision, and run-to-run reproducibility within acceptable analytical limits. Serum urea (Cat. No. 10505; 340 nm), creatinine (492 nm), alanine aminotransferase (ALT; Cat. No. 12012; 340 nm), aspartate aminotransferase (AST; Cat. No. 12011; 340 nm), and alkaline phosphatase (ALP; Cat. No. 12017; 405 nm) were measured using commercially available diagnostic reagent kits (HUMAN Diagnostics Worldwide, Wiesbaden, Germany). Serum total bilirubin was determined using the Jendrassik–Gróf colorimetric method (546 nm) with a commercially available diagnostic reagent kit (DiaSys Diagnostic Systems GmbH, Holzheim, Germany; Lot No. 60170070). All biochemical measurements were performed under standardized laboratory conditions. Enzyme activities were expressed as U/L**,** whereas serum urea, creatinine, and total bilirubin concentrations were expressed as mg/dL**.**

### 2.7. Histopathological examination

Liver and kidney tissues fixed in 10% neutral buffered formalin were processed using standard paraffin-embedding techniques. Tissues were dehydrated through ascending grades of ethanol, cleared in xylene, embedded in paraffin wax, and sectioned at 3–4 μm thickness using a rotary microtome. Sections were stained with hematoxylin and eosin (H&E) according to standard histological procedures. H&E-stained sections were examined and photographed using a light microscope equipped with a digital camera system. To ensure objective structural evaluation, all comparative histological images across control and treatment groups were captured under identical acquisition conditions, including uniform magnification (400x; 40x objective lens), constant illumination, identical exposure settings, and fixed white balance. Physical scale bars (50 μm) calibrated using an eyepiece micrometer were included directly on all representative micrographs to indicate structural dimensions. Histopathological evaluation was performed by an observer blinded to the treatment groups to minimize observer bias. Morphological alterations in hepatic and renal tissues were compared among the control and treatment groups.

### 2.8. Histopathological scoring

All H&E-stained liver and kidney tissue sections (one section per animal) were examined in their entirety by a single observer blinded to the experimental groups. Histopathological lesions were scored using the predefined semi-quantitative scoring system (0–4), and a single representative score was assigned to each tissue section.

#### 2.8.1. Liver histopathological scoring.

The severity of hepatic lesions was evaluated using a modified semi-quantitative histopathological scoring system (0–4) adapted from previously published toxicological studies, based on the extent of hepatocellular degeneration, sinusoidal dilatation, inflammatory cell infiltration, and disruption of hepatic architecture [23].

#### 2.8.2. Kidney histopathological scoring.

Renal lesions were evaluated using a modified semi-quantitative histopathological scoring system (0–4) adapted from previous experimental nephrotoxicity studies [24]. The scoring was based on the severity and extent of tubular epithelial degeneration and necrosis, loss of tubular brush borders, glomerular alterations, tubular dilatation, epithelial cell sloughing, interstitial inflammatory cell infiltration, and disruption of overall renal architecture.

**Table:** 

Score	Grading / Severity	% Area Affected	Liver Histopathological Features	Kidney Histopathological Features
0	Normal / None	0%	Intact hepatic lobular architecture, distinct central vein, uniform cords, no vacuolation or inflammation.	Normal renal architecture, intact proximal/distal tubules with preserved brush borders, healthy glomeruli.
1	Minimal / Mild	< 25%	Localized cytoplasmic clearing/vacuolation, minor sinusoidal expansion, isolated perivascular inflammatory cells.	Focal loss of tubular brush borders, mild swelling of tubular epithelial cells, sparse interstitial leukocyte infiltration.
2	Moderate	25% – 50%	Moderate hydropic change/vacuolation, multifocal sinusoidal congestion, perivascular inflammatory cuffing, scattered pyknotic nuclei.	Moderate tubular degeneration, sloughing of epithelial cells into tubule lumen, multifocal interstitial inflammatory infiltration.
3	Severe	50% – 75%	Widespread hepatocellular ballooning/degeneration, extensive sinusoidal dilatation, dense inflammatory aggregates, focal necrosis.	Severe tubular epithelial necrosis with loss of basement membrane, prominent tubular dilation with debris, dense interstitial infiltration.
4	Critical / Massive	> 75%	Massive loss of cord architecture, confluent area necrosis, severe vascular congestion, extensive parenchymal infiltration.	Diffuse/widespread tubular necrosis, ghost-like cellular outlines, extensive loss of organ structure, severe global inflammation.

### 2.9 Statistical analysis

Quantitative data are presented as mean ± standard error of the mean (SEM). Statistical analyses were performed using GraphPad Prism version 11.0 (GraphPad Software, Boston, MA, USA). Differences among experimental groups were analyzed using the Kruskal–Wallis test followed by Dunn’s multiple comparisons test. Histopathological scores were also analyzed using the Kruskal–Wallis test followed by Dunn’s multiple comparisons test. Pearson’s correlation analysis was performed to evaluate the relationships between serum creatinine and urea, and between serum alkaline phosphatase (ALP) and bilirubin. Scatter plots with fitted simple linear regression lines and 95% confidence intervals were generated to illustrate these relationships. A heat map based on row-wise Z-score normalization was generated to visualize relative biochemical alterations among the experimental groups. Statistical significance was set at *P* < 0.05.

## 3 Results

### 3.1 Biochemical analysis

Polystyrene microplastic exposure significantly altered both hepatic and renal biochemical parameters in mice (Fig 1; Tables 1–2). Serum ALT, AST, and ALP levels differed significantly among the experimental groups (Kruskal–Wallis, *P* = 0.0247, 0.0192, and 0.0044, respectively). Post hoc analysis showed that ALT, AST, and ALP levels were significantly elevated only in the high-dose (HD) group compared with the control group (ALT, *P* = 0.0288; AST, *P* = 0.0149; ALP, *P* = 0.0018), whereas no significant differences were observed between the control and low-dose (LD) or medium-dose (MD) groups (*P* > 0.05 for all comparisons). Serum bilirubin levels also differed significantly among groups (Kruskal–Wallis, *P* = 0.0045), with significantly higher concentrations in the HD group than in controls (*P* = 0.0032), whereas the LD and MD groups did not differ significantly.

**Table 1:** Serum biochemical parameters in mice following graded polystyrene microplastic exposure. Data are presented as mean ± SEM. Statistical analysis was performed using the Kruskal–Wallis test followed by Dunn’s multiple comparison test.

Parameters	Control	Low Dose (LD)	Medium Dose (MD)	High Dose (HD)	P-value
ALT	94.33 ± 14.61	146.5 ± 12.27	162.2 ± 11.94	201.3 ± 31.05	0.0247*
AST	94.83 ± 9.239	106.2 ± 10.09	123.0 ± 18.80	166.0 ± 13.63	0.0192*
ALP	176.2 ± 18.33	260.0 ± 26.75	271.7 ± 32.03	399.0 ± 37.79	0.0044**
Creatinine	0.5167 ± 0.1014	0.9667 ± 0.1308	1.300 ± 0.1880	1.517 ± 0.1327	0.0029**
Urea	28.65 ± 1.394	33.18 ± 2.298	48.17 ± 4.600	60.67 ± 3.955	0.0006***
Bilirubin	0.6167 ± 0.1014	0.9000 ± 0.1065	1.150 ± 0.1478	1.500 ± 0.1506	0.0045**

Statistical significance: ns = not significant; *P < 0.05; **P < 0.01; ***P < 0.001.

**Table 2:** Pairwise comparisons of serum biochemical parameters among experimental groups following graded polystyrene microplastic exposure. Statistical analysis was performed using the Kruskal–Wallis test followed by Dunn’s multiple comparison test.

Parameter	p-value	C vs LD	C vs MD	C vs HD	LD vs MD	LD vs HD	MD vs HD
ALT	0.0247*	0.6387 ns	0.0902 ns	0.0288*	>0.9999 ns	>0.9999 ns	>0.9999 ns
AST	0.0192*	>0.9999 ns	>0.9999 ns	0.0149*	>0.9999 ns	0.1397 ns	0.5869 ns
ALP	0.0044**	0.4716 ns	0.3430 ns	0.0018**	>0.9999 ns	0.3763 ns	0.5148 ns
Creatinine	0.0029**	0.5829 ns	0.0342*	0.0026**	>0.9999 ns	0.3743 ns	>0.9999 ns
Urea	0.0006***	>0.9999 ns	0.0422*	0.0009***	0.4743 ns	0.0255*	>0.9999 ns
Bilirubin	0.0045**	>0.9999 ns	0.1243 ns	0.0032**	>0.9999 ns	0.1710 ns	>0.9999 ns

Statistical significance is indicated as: ns = not significant; *P < 0.05; **P < 0.01; ***P < 0.001; ****P < 0.0001.

**Fig 1:**
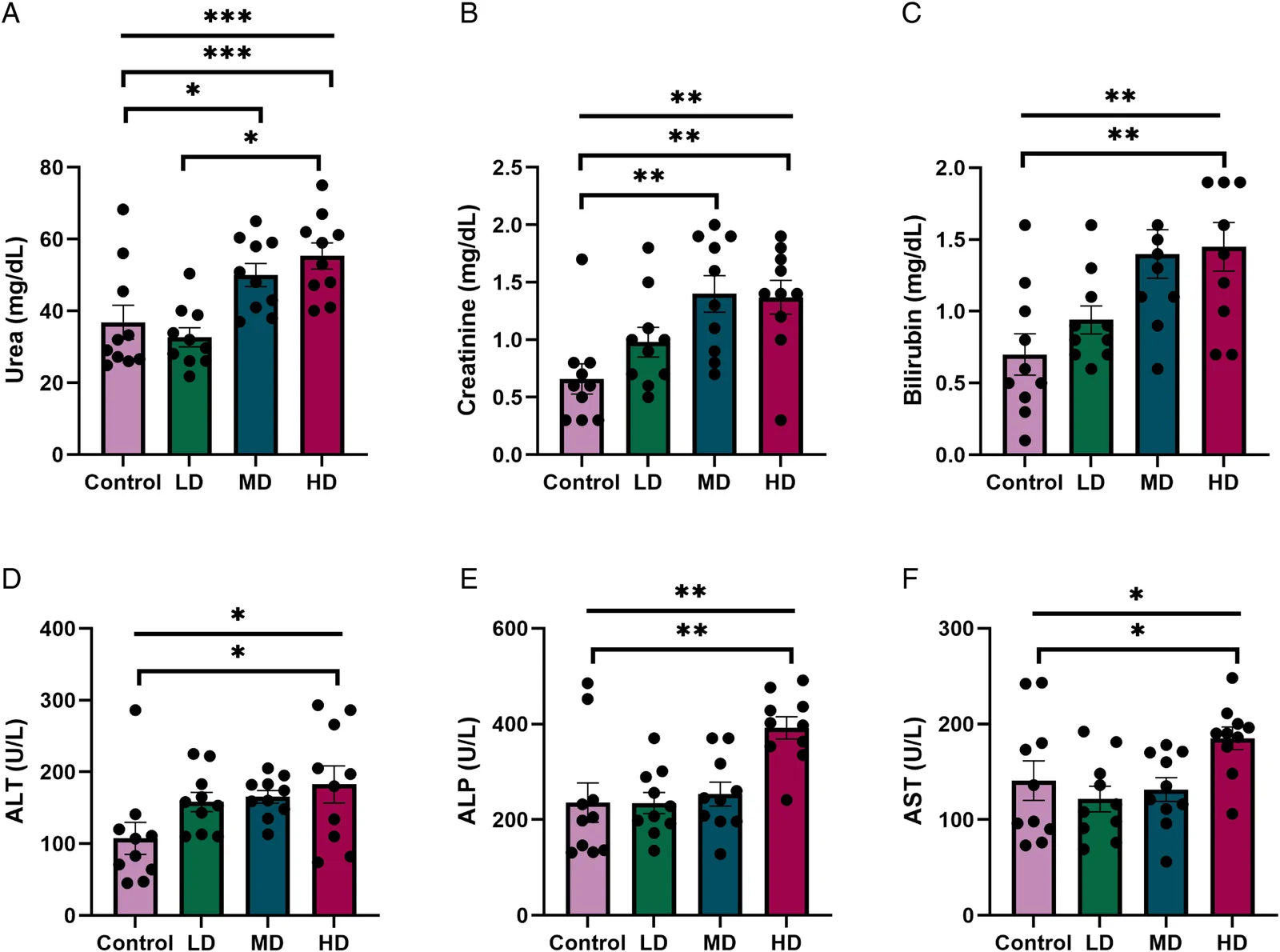
Effects of graded polystyrene microplastic exposure on serum biochemical parameters in mice. Serum levels of (A) urea, (B) creatinine, (C) total bilirubin, (D) alanine aminotransferase (ALT), (E) alkaline phosphatase (ALP), and (F) aspartate aminotransferase (AST). Bars and error bars represent mean ± SEM (n = 10 mice per group), with individual data values plotted as solid circles (n = 10). Overall group differences were analyzed using the non-parametric Kruskal–Wallis test, followed by Dunn’s post-hoc multiple comparisons test to determine pairwise statistical significance against the Control group or between treatment groups (indicated by horizontal comparison bars). Asterisks denote statistical significance derived from Dunn’s post-hoc test: ns = not significant, (P ≥ 0.05)*P < 0.05, **P < 0.01, ***P < 0.001, ****P < 0.0001.

Renal biomarkers also exhibited significant dose-related alterations. Serum creatinine levels differed significantly among the groups (Kruskal–Wallis, *P* = 0.0029), with significantly higher concentrations in the MD (*P* = 0.0342) and HD (*P* = 0.0026) groups than in controls, while the LD group remained comparable with the control group (*P* = 0.5829). Similarly, serum urea levels differed significantly among the experimental groups (*P* = 0.0006). Urea concentrations were significantly increased in the MD (*P* = 0.0422) and HD (*P* = 0.0009) groups compared with controls, and were also significantly higher in the HD group than in the LD group (*P* = 0.0255). No significant differences were detected between the LD and MD groups (*P* = 0.4743) or between the MD and HD groups (*P* > 0.9999).

Overall, the biochemical findings demonstrated a dose-related response to polystyrene microplastic exposure, with the most pronounced hepatic and renal alterations observed in the HD group.

### 3.2. Correlation analysis of biochemical parameters

Pearson’s correlation analysis revealed a strong positive correlation between serum creatinine and urea concentrations (r = 0.7628, R² = 0.5819, 95% CI: 0.5192–0.8918, *P* < 0.0001; Fig 2A). In addition, serum ALP levels were moderately positively correlated with bilirubin concentrations (r = 0.5229, R² = 0.2734, 95% CI: 0.1515–0.7649, *P* = 0.0087; Fig 2B). The corresponding correlation coefficients and statistical results are presented in Table 3.

**Table 3:** Pearson’s correlation analysis of selected serum biochemical parameters in mice exposed to PS-MPs. Correlations between serum creatinine and urea, and between serum alkaline phosphatase (ALP) and bilirubin, were assessed using Pearson’s correlation analysis.

Parameters	r-value	R^2^	95% Confidence Interval	P-value
Creatinine vs. Urea	0.7628	0.5819	0.5192–0.8918	<0.0001****
ALP vs. Bilirubin	0.5229	0.2734	0.1515–0.7649	0.0087**

Statistical significance is indicated as: ns = not significant; *P < 0.05; **P < 0.01; ***P < 0.001; ****P < 0.0001.

**Fig 2:**
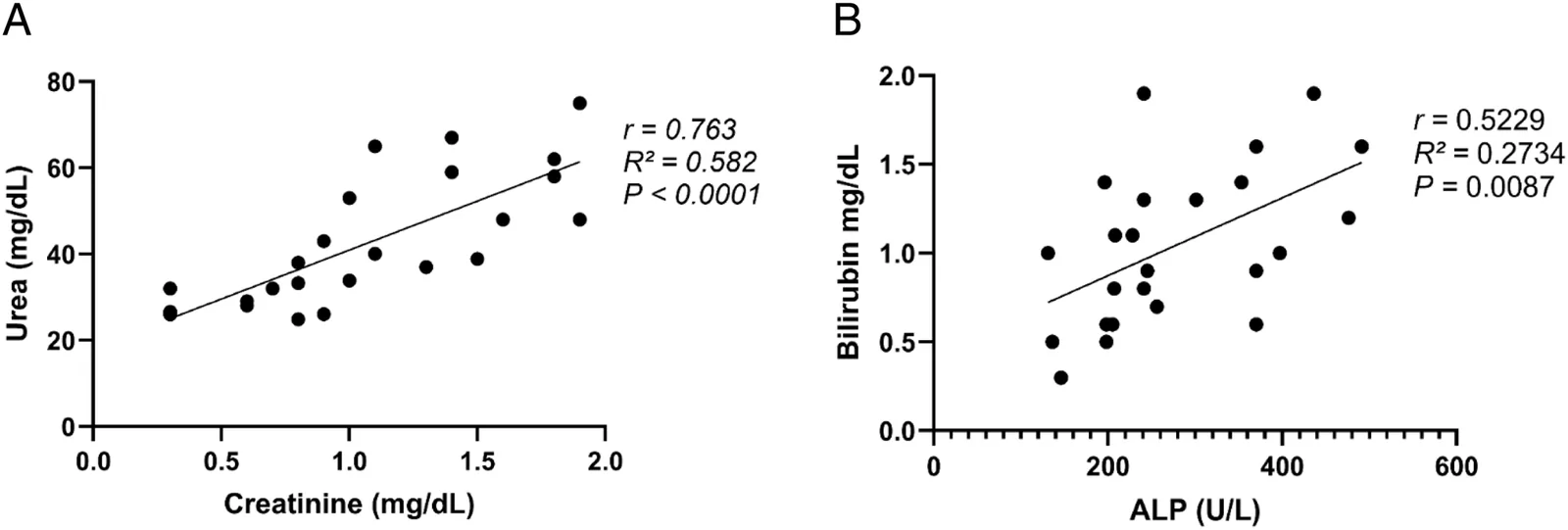
Pearson’s correlation analysis of serum biochemical parameters in mice (n = 40). (A) Correlation between serum creatinine and urea levels, showing a significant positive association (r = 0.7628, R² = 0.5819, P < 0.0001). (B) Correlation between serum alkaline phosphatase (ALP) and bilirubin levels, demonstrating a significant positive association (r = 0.5229, R² = 0.2734, P = 0.0087). The solid lines represent the simple linear regression fit for each analysis.

### 3.3. Heat map analysis

A heat map based on row-wise Z-score normalization illustrated the overall biochemical response to graded polystyrene microplastic exposure (Fig 3). The control group exhibited the lowest relative biomarker levels, whereas the HD group showed consistently higher values across hepatic (ALT, AST, ALP, and bilirubin) and renal (creatinine and urea) biomarkers. The LD and MD groups displayed intermediate profiles, reflecting a dose-related pattern of biochemical alterations.

**Fig 3:**
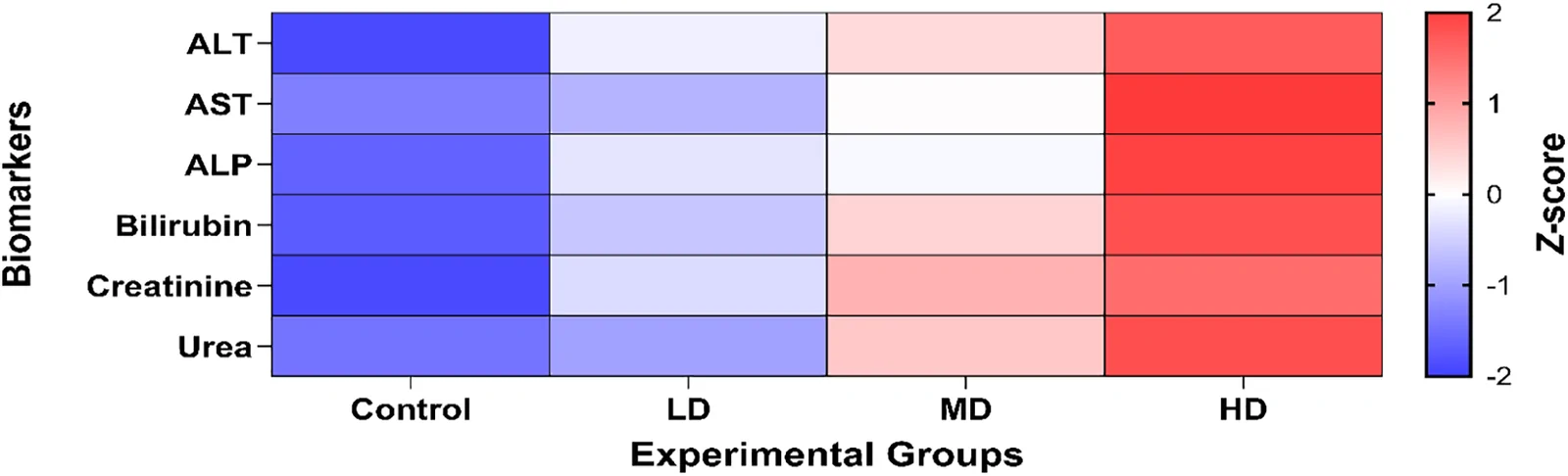
Heat map of row-wise Z-score-normalized serum biochemical parameters in mice following graded polystyrene microplastic exposure. Blue indicates lower values, white indicates values near the mean, and red indicates higher values.

### 3.4. Histopathological analysis

Representative hematoxylin and eosin (H&E)-stained liver and kidney sections demonstrated dose-related hepatorenal injury following polystyrene microplastic exposure (Figs 4–5). The control group exhibited normal hepatic and renal architecture. In contrast, the LD group showed mild hepatic lesions characterized by localized inflammatory cell infiltration, focal necrosis, sinusoidal dilatation, and hepatocellular vacuolizations, together with mild renal inflammatory cell infiltration and focal tubular epithelial necrosis. These alterations became more pronounced in the MD group, with extensive hepatic inflammatory infiltration, hepatocellular degeneration, multifocal necrosis, increased vacuolizations, and moderate renal tubular epithelial necrosis/degeneration. The HD group exhibited severe disruption of hepatic architecture, including diffuse hepatocyte vacuolizations, widespread sinusoidal dilatation and congestion, and extensive necrosis, accompanied by marked renal inflammatory cell infiltration and widespread tubular epithelial necrosis/degeneration.

**Fig 4:**
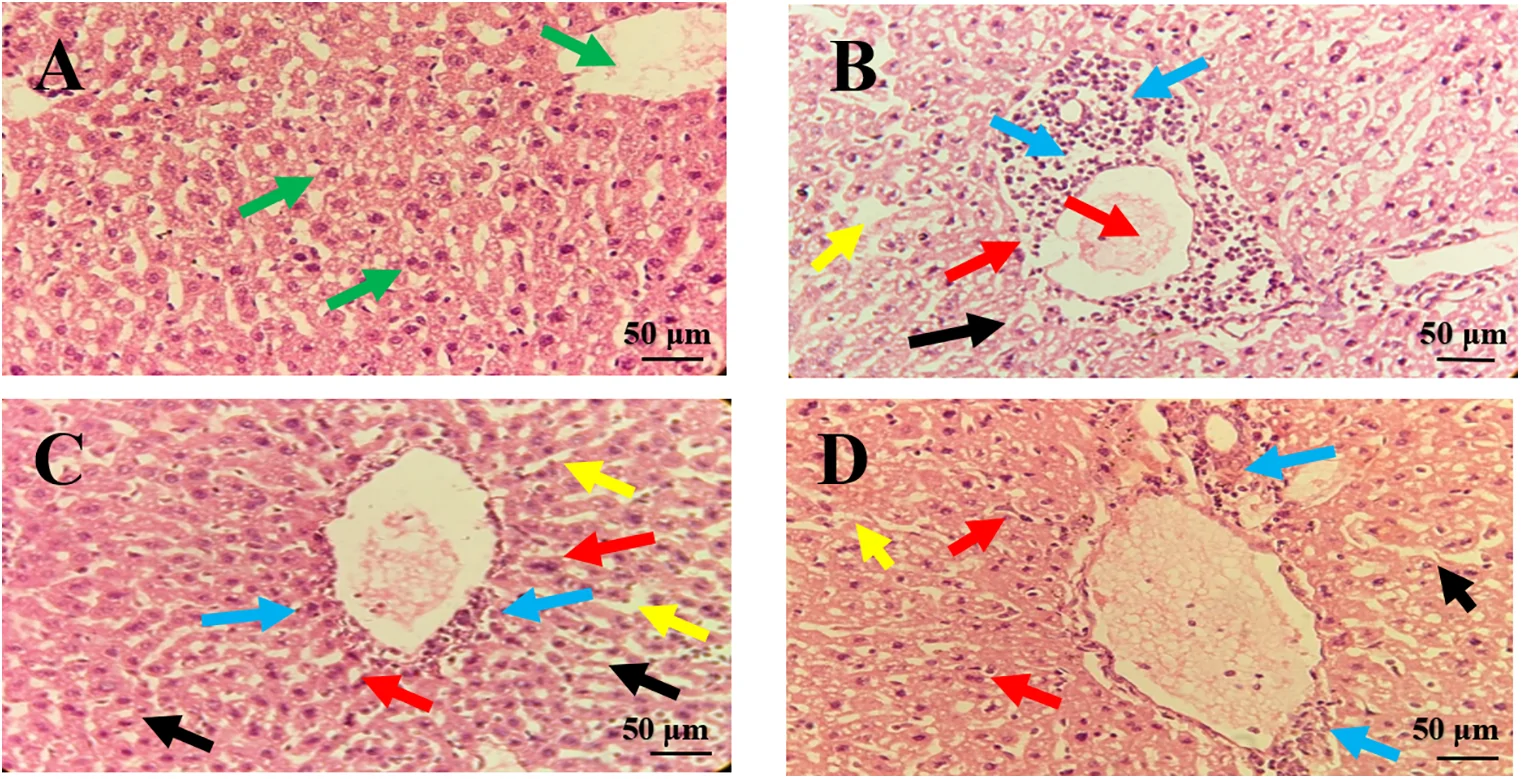
Representative H&E-stained liver sections from mice following graded polystyrene microplastic exposure (400 × ; scale bar = 50 μm). (A) Control; (B) low-dose (10 mg PS-MPs/kg body weight/day); (C) medium-dose (20 mg PS-MPs/kg body weight/day); and (D) high-dose (30 mg PS-MPs/kg body weight/day). Green arrows indicate normal hepatic architecture; blue arrows, inflammatory cell infiltration; red arrows, necrosis; yellow arrows, sinusoidal dilatation; and black arrows, hepatocyte vacuolizations.

**Fig 5:**
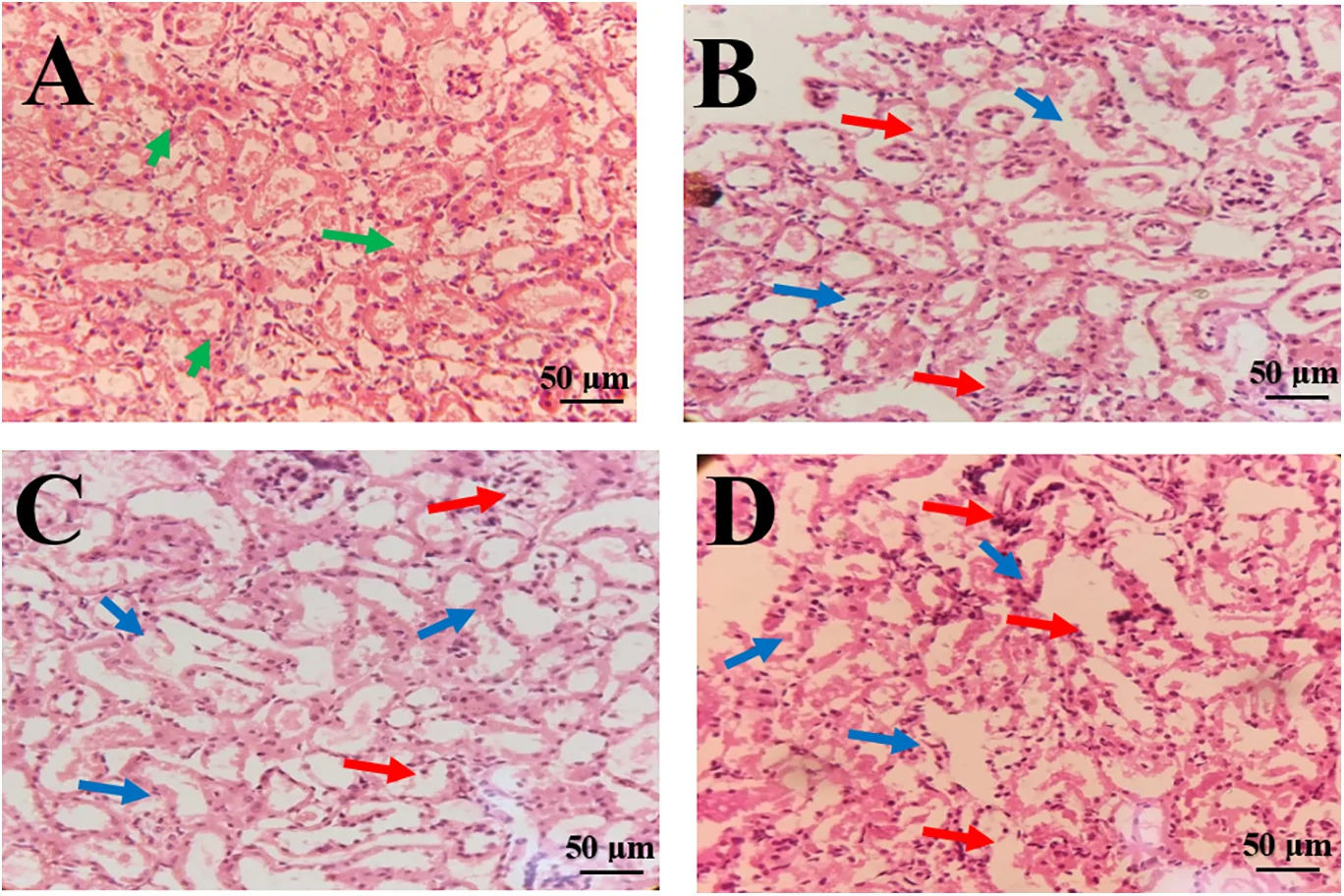
Representative H&E-stained kidney sections from mice following graded polystyrene microplastic (PS-MPs) exposure (400 × ; scale bar = 50 μm). (A) Control; (B) low-dose (10 mg PS-MPs/kg body weight/day); (C) medium-dose (20 mg PS-MPs/kg body weight/day); and (D) high-dose (30 mg PS-MPs/kg body weight/day). Green arrows indicate normal renal architecture; blue arrows, inflammatory cell infiltration; and red arrows, tubular epithelial necrosis/degeneration.

Semi-quantitative histopathological scoring confirmed significant differences among the experimental groups (Kruskal–Wallis, *P* < 0.0001; Fig 6). Hepatic inflammatory cell infiltration scores were significantly increased in the MD (*P* = 0.0007) and HD (*P* < 0.0001) groups compared with controls, whereas the LD group remained comparable (*P* = 0.7626). Similarly, renal inflammatory cell infiltration and tubular epithelial necrosis/degeneration scores were significantly elevated in the MD (*P* = 0.0005 and *P* = 0.0004, respectively) and HD (*P* < 0.0001 for both) groups, while no significant differences were observed in the LD group (*P* > 0.05). Histopathological scores increased progressively with increasing microplastic dose, with the most severe lesions observed in the HD group.

**Fig 6:**
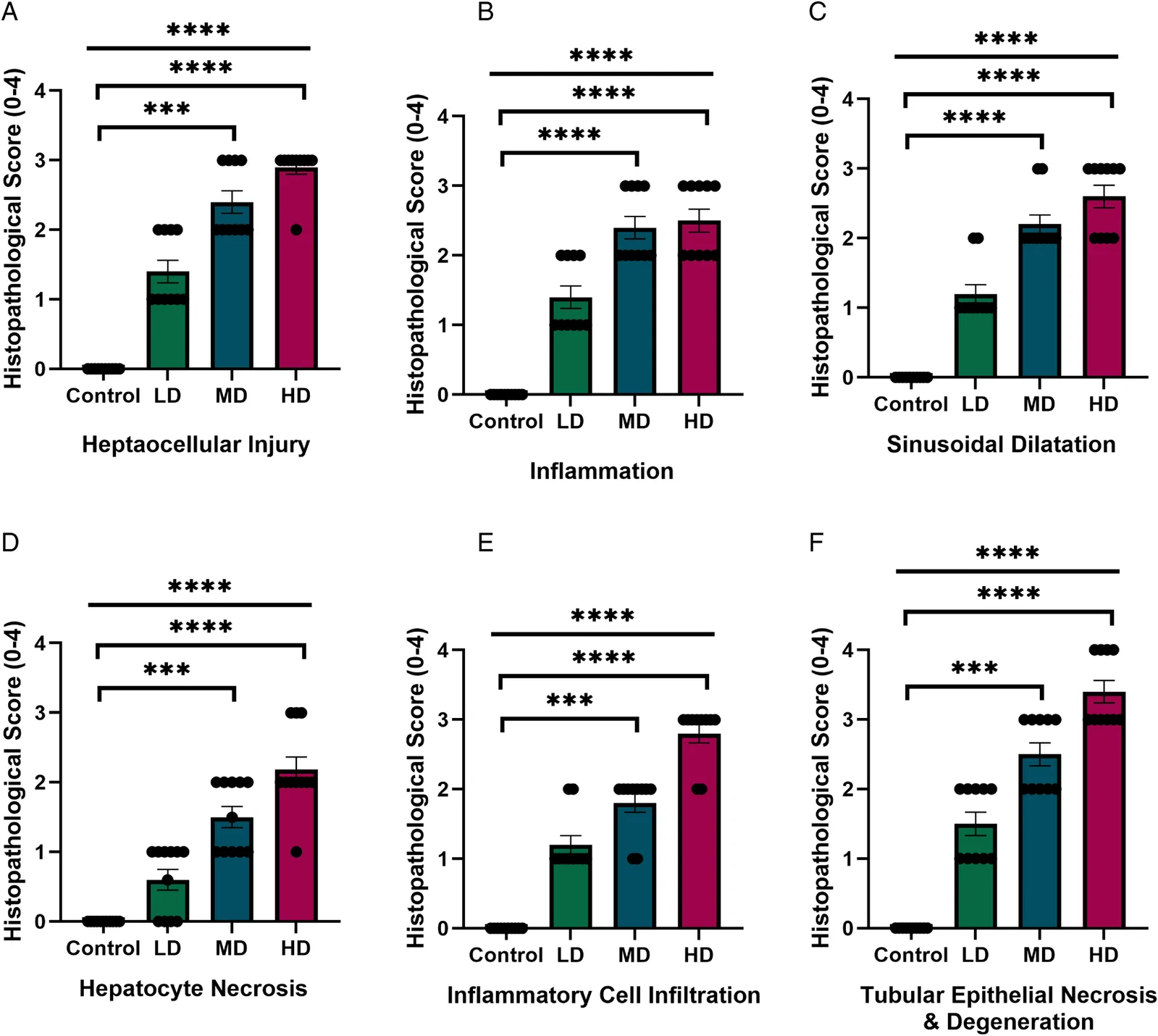
Semi-quantitative histopathological lesion scores of liver and kidney tissues in mice following PS-MP exposure. Ordinal histopathological scores (range 0–4) evaluating hepatic lesions a) hepatocellular injury, b) inflammation, c) sinusoidal dilatation, d) hepatocyte necrosis and renal lesions e) inflammatory cell infiltration and f) tubular epithelial necrosis and degeneration). Bars represent mean ± SEM (n = 10 mice per group) overlaid with individual mouse scores shown as solid circles to reflect ordinal score distribution. Group comparisons were performed using the non-parametric Kruskal–Wallis test followed by Dunn’s post-hoc multiple comparisons test for pairwise evaluation against the Control group (indicated by horizontal comparison bars). Asterisks represent statistical significance determined by Dunn’s post-hoc test: ***P < 0.001, ****P < 0.0001.

## 4. Discussion

The present study demonstrates that exposure to polystyrene microplastics (PS-MPs) was associated with biochemical and histopathological alterations in the liver and kidneys of BALB/c mice. The increases in serum ALT, AST, ALP, bilirubin, urea, and creatinine, together with tissue alterations, indicate changes in hepatorenal function following oral PS-MP exposure.

The elevation of hepatic biomarkers observed in the present study indicates altered hepatocellular injury following PS-MP exposure. Increased serum ALT and AST, together with elevated ALP and bilirubin, suggest impairment of both hepatocellular integrity and hepatobiliary function. These findings are consistent with previous reports demonstrating the hepatotoxic effects of PS-MPs, including oxidative stress, metabolic disturbances, hepatic inflammation, altered lipid metabolism, and inflammatory responses [8,10,25]

The histopathological findings further support these biochemical changes, with inflammatory cell infiltration, hepatocyte vacuolization, necrosis, and sinusoidal dilation showing greater severity at higher exposure levels**.** These observations are compatible with previous studies showing that PS-MPs impair hepatic antioxidant capacity and induce structural abnormalities in liver tissue. Chronic exposure to polystyrene particles has also been associated with hepatic inflammation and metabolic disturbances [11,26]. Collectively, these findings suggest that PS-MP exposure compromises hepatic architecture, likely through oxidative injury and inflammatory signaling.

Renal effects were evident from increased serum creatinine and urea, particularly in the medium- and high-dose groups. These alterations indicate changes in renal function, while the strong positive correlation between creatinine and urea further supports their association with renal functional changes. Previous studies have reported that microplastic exposure can impair renal antioxidant defenses, stimulate inflammatory pathways, and induce oxidative injury [12,27]. In the present study, these functional changes were accompanied by inflammatory cell infiltration and tubular epithelial degeneration/necrosis, providing morphological support for renal involvement. Similar structural changes have been reported following PS-MP exposure [11].

The greater biochemical and histopathological changes observed at higher exposure levels suggest that exposure concentration may influence the severity of PS-MP-associated toxicity. While low-dose exposure produced relatively mild alterations, the medium- and high-dose groups showed more pronounced changes. The present pattern is broadly in accordance with previous experimental evidence of concentration-related effects following microplastic exposure [6,8].

The significant positive correlation between ALP and bilirubin further supports their association with liver-related changes. Although correlation analysis does not establish causality, the concurrent elevation of these biomarkers suggests changes in liver function following PS-MP exposure. Similar changes in liver enzymes and bilirubin have been reported following exposure to environmental contaminants, supporting the possibility that microplastics may contribute to liver-related effects through multiple interacting mechanisms [7].

Several limitations should be acknowledged. Only male mice and a 28-day exposure period were used, limiting generalizability and assessment of long-term effects. Molecular mechanisms, oxidative stress, inflammation, and PS-MP accumulation were not investigated. Future studies should include both sexes, longer exposures, environmentally relevant doses, and mechanistic analyses.

## 5. Conclusion

The present study demonstrates that 28-day oral exposure to 5 μm PS-MPs is associated with hepatorenal toxicity in mice, characterized by biochemical alterations and histopathological injury that generally increased with exposure dose**.** The principal contribution of the study is the integrated assessment of hepatic and renal effects using complementary biochemical and histopathological endpoints across graded PS-MP exposure levels**.** Further studies using longer exposure periods, environmentally relevant concentrations, and mechanistic approaches are warranted to better understand the health implications of PS-MP exposure.

## Supporting information

S1 File Supplementary data. This file contains the raw experimental data and statistical analyses supporting the findings of this study.
(XLSX)

S1 Fig Graphical Abstract. (TIF)
